# ‘Pilot RCT of a new treatment for child conduct problems that have not responded to evidence-based parent training’

**DOI:** 10.1371/journal.pone.0353611

**Published:** 2026-07-28

**Authors:** Helen Sharp, Hermione Roff, Nicky Wright, Andrew Pickles, Jonathan Hill

**Affiliations:** 1 Department of Primary Care and Mental Health, University of Liverpool, United Kingdom; 2 School of Psychology, Manchester Metropolitan University, United Kingdom; 3 Department of Biostatics & Health Informatics, King’s College London, United Kingdom; 4 Oxford Health NHS Foundation Trust, United Kingdom; Kalinga Institute of Medical Sciences, INDIA

## Abstract

**Background:**

Children with conduct problems are at high risk of persisting mental health problems, making them a priority for early intervention. Group-based parent-training is effective but with a substantial failure rate. Based on evidence on the value of involving children, we developed Reflective Interpersonal Therapy for Children and Parents, (RICAP). We report feasibility and outcomes from the first pilot trial of an intervention for children with conduct problems persisting after parent training. In contrast to most studies, we used both parent and teacher report.

**Methods:**

The sample comprised 105 children and their parents aged 4–10 years inclusive referred to UK Child and Adolescent Mental Health Services (CAMHS) with conduct problems. All were offered the Incredible Years (IY) parent training intervention, and parents provided pre- and post-treatment measures (including CBCL, SDQ). Children still above clinical threshold after IY were randomized either to RICAP or to usual CAMHS treatment (CTAU) with follow up 8 months later. Only those randomized to CTAU could receive school-based interventions. Trial Registration Number: ISRCTN25252940.

**Results:**

Feasibility was supported by high retention through the initial IY (102/105) and subsequent RCT phases of the study (58/70 eligible for randomization). The majority of those randomized to RICAP attended for 11/14 or more sessions, reflecting its high acceptability to both children and parents. By parent report RICAP was superior to CTAU on CBCL externalising (d = 0.32) and internalising (d = 0.42) problems, while by teacher report CTAU was superior on SDQ total problems (d = 0.32) and reactive aggression (d = 0.27).

**Conclusions:**

We provide first evidence of the feasibility of a design in which non-responders to parent training are randomized to novel intervention or control conditions. Outcomes were different for parent and teacher reports, highlighting for future randomized controlled trials the need to attend to possible reporter and treatment context variations.

## Introduction

Disruptive behavioural disorders are among the most prevalent disorders in childhood, with 5% of British children meeting International Classification of Disease (ICD-10) criteria for conduct disorder and oppositional defiant disorder (ODD) [1] and global prevalence estimated to be 5.7% [2]. Multiple agencies are involved in responding to these difficulties, with a high proportion of children presenting to GP and emergency services regularly with conduct related issues [3]. Antisocial, disruptive or aggressive behaviour is a presenting problem in 30% of a typical GP’s child consultations and 45% of consultations in community child health [4]. In England, individuals with conduct disorder in childhood cost society ten times as much as control children by adulthood [3] and in the United States the lifetime cost to the public of conduct problems was estimated at $2.3 million [5].

Numerous longitudinal general population studies have confirmed that disruptive behavior problems seen in young children commonly persist. They are associated with increased risks not only for antisocial and poor interpersonal outcomes, but also for many psychiatric disorders, notably, depression, anxiety disorders, PTSD, and alcohol and drug misuse [6,7]. Equally, in spite of this markedly elevated risk, the majority of children with early onset disruptive behavior problems (typically estimated at between 50–70%) do not show persistence of those problems into adult life. However, even those whose behaviours have improved have worse mental, physical, social and occupational outcomes compared to children with low lifetime problems [8,9].

For those with the “life-course persistent” problems, outcomes are commonly very poor. In adolescence these children are at increased risk of poor peer relationships, exclusion from education, higher risk of unemployment, and involvement in criminal activity [10,11], depression [12,13] and suicidality [14]. In adulthood, poor outcomes include features of antisocial personality disorder such as criminality and poor interpersonal and occupational functioning. The risks also extend to a wide range of disorders, notably depression and drug and alcohol misuse, and to partner violence, negative parenting and physical abuse and hence risk to the next generation [7,8,15,16]. Children of antisocial adults are more often exposed to harsh and inconsistent parenting, low warmth, and reduced parental sensitivity, each of which is strongly associated with the onset and maintenance of child behavioural problems [17–19].

The UK National Institute for Clinical Excellence [4] recommend the use of manualised group-based parent training interventions such as the Incredible Years (IY) video-based parent training programme [20] as a first line treatment response for individuals referred with ODD or conduct problems. Informed by behavioural and social learning theories, such interventions aim to address maladaptive patterns of reinforcement of unwanted behaviours and are efficacious in specialist research settings [21,22], in ‘real world’ child and adolescent mental health services [23] and in Sure Start or voluntary sector settings to prevent conduct problems in preschool children [24,25]. However, a sizeable proportion of families drop-out prematurely or do not attend group interventions (30–50%) and a substantial number of children remain in the clinical range following treatment [3,26–28]. A major concern is that factors known to predict poorer outcome in parent training also predict persistence of antisocial behaviour in longitudinal studies. These include socio-economic deprivation, severity of child conduct problems, parental mental health problems, parental unresolved loss or trauma and partner violence in the home [1,29–32]. So, a serious challenge remains to develop interventions that address the needs of these families. Scott and Dadds [28] propose that a multi-model approach incorporating strategies based on alternative theoretical approaches such as attachment theory, structural family systems theory and cognitive-attribution theory may enhance the effectiveness of parent training.

Although, as described in the previous paragraph, the drop-out and non-response rates for group-based parent training are well documented, there have been no previously published evaluations of interventions designed to help when clinically significant conduct problems persist despite parent training. In the context of how to help where parent training has not led to substantial improvement, one of the recurring views expressed by parents is that the particular needs of their children have not been addressed [33]. As a result, aspects of the child that contribute to problem behaviours, such as limited empathy, difficulties in recognizing their own emotions, underlying anxiety, or other differences among children, may be neglected. Addressing individual child vulnerabilities may therefore be key. An extensive research literature shows that individual child vulnerabilities are strongly associated with persistent forms of antisocial behaviour and these characteristics may also limit the success of interventions that are confined to working with parents. Such vulnerabilities include limited verbal skills, pragmatic language problems, deficits in executive function and information processing biases contributing to misperception of hostile cues from others and to inflated self-appraisal of competence [34–36]. Insecure parent-child attachment relationships are also significantly over-represented, and these are associated with child difficulties in interpreting and responding to other people’s emotions particularly when under conditions of emotional threat [10,37]. Another set of vulnerabilities characterise a distinct group of children with conduct problems who also show callous-unemotional (CU) traits. CU traits include a lack of guilt, empathy and concern for others, unconcern about performance and shallow affect [38]). Children with CU traits experience greater conduct problem severity and higher absolute rates of symptoms after treatment compared to children without CU traits [39].

There are therefore good reasons to include children in interventions designed to improve outcomes for those not substantially helped by parent training. Several child focussed approaches have been developed and evaluated. Social skills training for children with disruptive behaviour problems has been shown to be effective, albeit with small effects [40]. The Coping Power Program (CPP) combines work with the child and parent training. The child approach uses a cognitive-behavioural framework to enhance the child’s ability to cope adaptively with difficult situations and feelings [41]. These interventions were originally designed to prevent the development of antisocial outcomes in children at risk, but more recently have been adapted to clinical samples in Sweden, Netherlands and Italy [42–44]. There is evidence that the effectiveness of CPP is sustained over up to six years [45]. However, to our knowledge, neither of these approaches has been evaluated as treatment for parent-training non-responders.

In designing the intervention for this study, Reflective Interpersonal Therapy for Children and Parents (RICAP), we took account of the considerable heterogeneity within the conduct disorders [10,46,47]. This has been characterised in several ways, but a prevailing theme across several proposed typologies is the contrast between reactive anger prone opposition, and proactive more emotionally neutral or cold, low empathy, disruption or aggression [48–52]. We also took account of the possible role of anxiety in driving some oppositional or aggressive behaviours. While evidence for this is limited, a key hypothesis for the developmental consequences of disorganized attachment is that the child creates their own organisation through controlling behaviour, and importantly for RICAP ‘controlling-punitive’ behaviours [53, 54]. The controlling behaviours are thus an attempt to deal with anxieties for which the child does not turn to the parent. Crucially, the insecurity and anxieties may not be apparent to the caregiver, because only the disruptive behaviours are apparent. RICAP therefore aims to create an arena in which the child’s anxieties can be identified and understood by the parent leading to changes in parental behaviour.

Enhancing a child’s reflective capacity, that is their awareness and understanding of their own and other’s thoughts and feelings, was in our view also a candidate for the focus of the intervention. Increased reflectiveness would create opportunities for children to modify social cognitions associated with anger proneness [34] to respond empathetically to others, and also to recognise their own underlying feelings, learn new ways of expressing them and to make new choices for interpersonal behaviour as a result. Finally, the development of RICAP was also informed by findings from our research group, using story stems in the context of doll play. In response to emotionally challenging doll-play scenarios, children who demonstrated or described in their play the motives of the doll participants, their ‘intentionality’, had lower levels of disruptive behaviour problems [37,55]. This further suggests that an intervention designed to increase child reflective functioning within interpersonal exchanges may be helpful.

The RICAP method contains some elements of structure (e.g., certain topics for drawing are introduced) but it is flexible enough to ensure a personalised approach. Table 1 gives a summary of the aims and components within the intervention. The approach with the child makes use of both the representational content and process of child drawing to help identify attachment related concerns and social-cognitive or attributional biases in the child’s view of the world. The therapist-child conversation in session is designed to promote an autonomous capacity to reflect and self-reflect, to broaden the child’s understanding of self and others in interpersonal exchanges. This in turn opens up new possibilities for adaptive emotional expression and alternative child behavioural choices. A book is compiled by the child therapist to record the drawings and conversations from each session. Since many of these difficulties manifest themselves within the parent-child relationship, we developed a parallel approach with parents designed to increase their own reflective capacity about self and other. This includes understanding the range of underlying feelings and motivations that might underpin their child’s opposition, defiance or aggression in specific circumstances that occur day to day. The method uses curious questioning to tease apart in detail the sequence or chain of events that occurred in the parent-child interactions during a good time and difficult time which the parent chooses from the past week. The aim was to help parents build new understandings of their child and generate a broader repertoire of possible responses in the light of those understandings, so they might feel able to make more informed choices about how to respond in a given situation. These new understandings then have the potential to increase parents’ ability listen to their child, to respond empathically and consistently [56,57] whilst also setting appropriate behavioural limits when required.

**Table 1:** Reflective Interpersonal Therapy for Children and Parents (RICAP) description.

Reflective Interpersonal Therapy for Children and Parents (RICAP)	
*Aim* The aim for child: to facilitate an autonomous capacity to reflect and self-reflect and to act appropriately on that reflection. The aim for parent: to understand their *child’s* understanding of events and relationships and to make choices about their interactions within those understandings.	*Practical overview* RICAP is a brief intervention (14 sessions) that is concurrent for parent and child, reflective, interactive, and practical. Sessions comprise 1 set-up (at the start) and 1 review session (at the end) with two therapists, parent(s) and child to establish aims and commitment and review progress together respectively. In the middle there are 12 individual weekly child sessions and 6 parallel fortnightly parent sessions. The parent also joins in the first and last 10 minutes of each child session (60 minutes) – see focus of parent and child sessions below. In between sessions the two therapists discuss and revise the formulation regarding each family after each session and use hypotheses generated to inform reflections within subsequent sessions. Both method and process promote change.
*Particular emphasis is placed on addressing the individual’s vulnerabilities:* - Impaired verbal ability, especially for emotions – aim to expand repertoire for interpreting and responding to emotions in interpersonal interactions. - Impaired executive function – aim to increase repertoire of ideas about solutions. - Apparently inflated self-appraisal – aim to identify fragile self-concept, support genuine sources of pride.	- Insecure attachment – aim to identify accurately attachment concerns and ways of expressing them and encourage development of new ways to meet these needs. - Promote self-reflective account of interactions - Identify sources of anxiety and adaptive solutions - Creates a basis for behavioural change
*Work with the child:* - Aim to provide the child with enhanced resources for understanding and responding to social challenges thereby enabling them to develop their own alternatives to oppositional behaviours or violence. - The focus is on facilitating the child’s capacity to reflect and self-reflect. - Therapist style is reflective, drawing out possible meanings from children’s drawings, is boundaried in the face of challenges and opposition, and behaviourally directive with child in session as appropriate. - The therapist and child progress through a series of structured topics co-creating a book with drawings and a written record of their conversations, reflections and understandings. Initial sessions focus on specific requests: ‘Draw yourself’ (Session 1), ‘Draw who you think of when you think of your family’ (session 2), ‘Draw you and your mum doing something together’ (Session 3), ‘Draw the problem’ (session 4) graduating to ‘Draw what’s on your mind’ which is the topic for the remaining sessions. These drawings can be reconsidered and revised as the treatment progresses. The conversational narrative of each session and the themes that arise are thus dependent on the child’s drawing and the therapist’s reflections and conversations with the child, and their co-constructed emerging understandings. - The therapist compiles the book in between sessions from the content of the session, and the child checks over and verifies the book at the beginning of the next session, with complete freedom to challenge, discuss or record anything he/she disagrees with. - The book is a holder of memory. The parent joins for 10 minutes at the beginning and end of each session to review specific events during the past week and plan together for the next week.	*Work with the parent:* - Parallel work with a parent by another therapist in six fortnightly individual sessions. - Designed to promote parental reflectiveness about the child’s thoughts and feelings, and hence promote making different choices about their interactions with their child. - Discussion focuses on specific examples of events and behaviours in the past week. The parent brings one good time and one difficult time with their child in the past week to each session. - Their task is to understand their *child’s* understanding of the times they bring, thus thinking about the events from the child’s point of view, making links between thoughts, feelings and behaviour. - A therapeutic letter is written by the therapist to the parent after each of these sessions as a record of their conversations and to record developing understandings of the child and any plans for new parental behavioural responses in the light of new understandings. - Emphasis is on generating multiple hypotheses about the meaning of child behaviour through reflection and self-reflection regarding child feelings, thoughts and behaviours, not on generating a ‘correct’ understanding. - The letter represents a detailed, ‘slowed down’ record of each incident. It presents different, multiple perspectives and understandings of those incidents. It generates an understanding of the interplay of personalities and of people in the relationship, the link between inner emotions and outer actions. It represents a record of the progress of the parent’s understanding of their child’s understanding and a written context within which change can be experimented with and choices for parental responses can be made.
*Framework* RICAP provides a framework for the child in dealing with their avoidance and aggressive actions in a safe and containing way. It provides a framework for the parents for looking at their avoidance in thinking about their child, that is both challenging and supportive. It provides a framework for the therapist in shaping and moulding understanding, and moving that forward, through reflection and self-reflection, into relationships and interactions.	

The overarching logic model for change in RICAP includes both child and parent processes. Increased awareness of their own thoughts and emotional states, and those of others, provides children with alternative coping strategies. It also supports more accurate communication of their needs to their parents. The child can also make new behavioural choices in the light of their own new understandings. More accurate communication to parents in turn makes it more possible for a parent to match their responses to the child’s needs. The parent’s increased reflectiveness enables them to go beyond the immediate behaviours of opposition and aggression, to consider a range of alternative drivers and hence broaden their repertoire of responses. Thus the parent is able to refine their responses, for example responding firmly when guidance is required, and tenderly where comfort and understanding are needed. Change also follows from the parent’s effective and accurate responding in these different domains of parent-child interaction.

In summary, ‘Reflective Interpersonal Therapy for Children and Parents’ (RICAP [56]) is a standardised and at the same time flexible intervention for conduct problems designed to address individual child and parent vulnerabilities. RICAP aims to enhance childrens' and parents’ awareness of thoughts and feelings predominantly in the parent-child relationship, in order to provide a platform for alternative less conflictual or hostile interactions.

We report results from a pragmatic pilot randomised controlled trial (RCT) in which children rated in the clinical range for conduct problems either by parents or by teacher at the end of a ‘gold standard’ parent training intervention (The IY programme [20]) were randomised to receive RICAP or clinical treatment-as-usual (CTAU). There were three main aims of the study. The first was to assess feasibility of the design within UK NHS settings, and in particular to determine retention across a study comprising an initial phase of IY intervention for all participants followed by randomization of children with persisting behaviour problems. The second aim was to find out whether families not helped by a previous intervention took up, and stayed in, the new treatment, RICAP. The third was to generate effect sizes from a range of outcome measures in order to inform future full trial design.

## Methods

### Study population

Eligible children were between the ages of 4 years and 10 years 11 months, referred for child conduct problems to Child and Adolescent Mental Health services (CAMHS) or multiagency community behavioural services (Behaviour Education Support Teams) on Merseyside, UK. These services were identified because they had experienced therapists routinely offering the IY Programme to groups of parents as a first line intervention. Participants were required to be English speaking.

The criterion for inclusion in the study was a score in the clinical range on either the CBCL/TRF externalising subscale or SDQ conduct problems subscale from parent and/or teacher informant (see Measures). Children with a prior diagnosis of ADHD were eligible if not receiving medication or if on an established dose of stimulant. Ethical approvals were gained from the Liverpool Children’s Research Ethics Committee and Wirral Health Authority Research Ethics Committee (REF 03/05/051/C) and written informed consent was obtained from all parents. The sample size calculation is shown in Supplementary Materials 1.

### Design

A two-phase pragmatic pilot RCT (Trial Registration Number ISRCTN25252940 https://www.isrctn.com/ISRCTN25252940). The trial was not registered prior to the start of recruitment because at that time, September 2003, the International Committee of Medical Journal Editors (ICJME) requirement for trial registration had not been introduced.

In Phase 1 all parents who consented to take part in the study, and met the inclusion criterion were offered group-based IY parent-training. Following the Phase 1 parenting-training, at 4 months, families with children rated in the clinical range either on parent or teacher reported CBCL, TRF or SDQ were eligible for randomization either to RICAP or CTAU in Phase 2.

Randomization was done with allocation concealment. A block randomisation design was used, using blocks of six to ensure similar sample sizes in each arm. Following baseline assessment, a researcher was told the allocation by an administrator sitting in a different building who had no knowledge of the study design. The administrator obtained the allocation from a randomisation schedule, devised by an independent statistician. Measures were administered prior to Phase 1 (Time 1, Baseline), 4 months later (time 2 – post phase 1 treatment and pre phase 2 treatment) and 12–14 months after baseline (time 3- post phase 2 treatment). Time 3 measures were gathered by research assistants who had not carried out previous assessments with the family, blind to treatment allocation. Teachers were not informed of the children’s treatment allocation. All families were followed up at one year after initial recruitment regardless of whether they had received a phase 2 intervention or not. At the point of consent parents were informed that the study was designed to compare ‘two different ways of working with parents and children’ after parent training. They were not informed that one was a novel therapeutic approach so as to minimise expectancy effects. The instructions given to participants are shown in Supplementary Materials 2. Recruitment began on 04/09/2003 and the trial ended on 21/09/2006.

**Fig 1:**
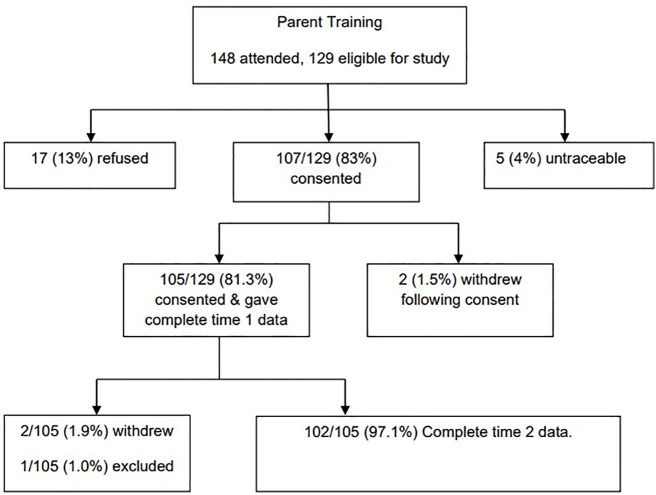
CONSORT diagram for recruitment and retention to Phase 1 parent training (IY) for all families.

**Fig 2:**
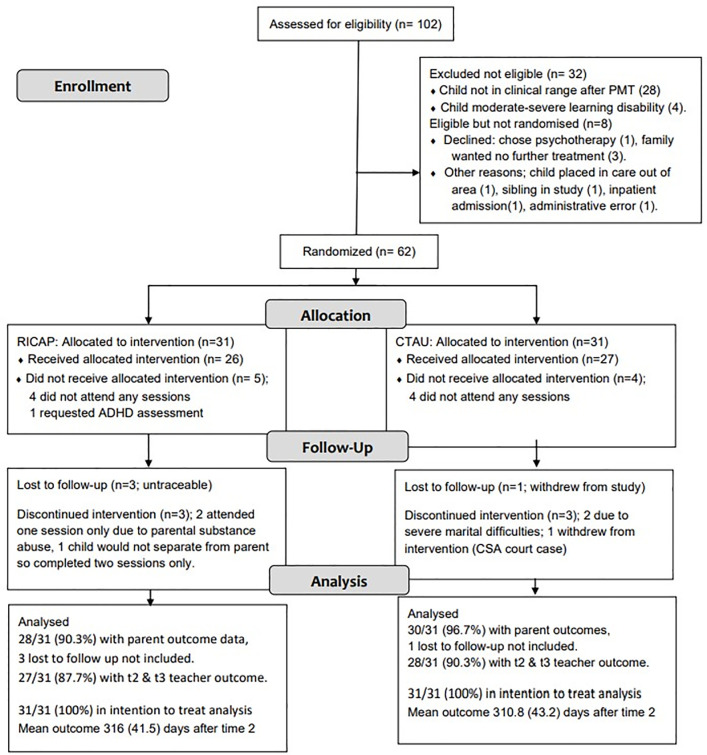
CONSORT diagram for recruitment to Phase 2 randomization to either RICAP or CTAU, and retention at follow up.

The study was designed according to the MRC guidelines on Phase Two Exploratory Studies of complex interventions (MRC, 2000) and the CONSORT statement [58,59]. The trial details are reported in line with the CONSORT guidelines for reporting of parallel, pragmatic and non-pharmacological interventions [60] which recommend the inclusion of a checklist, shown in the supplementary materials (S1 File), and CONSORT flow diagrams, which are shown for each phase of the study (Figs 1 and 2).

### Measures

#### Child Behaviours.

The parent report version of the Child Behaviour Checklist (CBCL [61]) and the Teacher Report Form (TRF [62]) were administered to assess externalising, internalising and total problems at Time 1, Time 2, and Time 3. Internal reliability (Cronbach’s alpha) was consistently high: Time 1 (parent report α = .81; teacher report α = .85), Time 2 (parent report α = .82; teacher report α = .84) and Time 3 (parent report α = 87.; teacher report α = .85).

The Strengths and Difficulties Questionnaire (SDQ [63]) parent and teacher report was also used to assess conduct problems and total problems, because it is widely used in epidemiological studies. It also showed good internal reliability: at Time 1 (parent report α = .69; teacher report α = .75), Time 2 (parent report α = .74; teacher report α = .77) and Time 3 (parent report α = 72.; teacher report α = .78).

A Parent Daily Report (PDR [64]) was gathered at two time points. This measure records 31 problem behaviours as present or absent each day for up to 7 days. This was administered over the telephone for a 1–2 week period at Time 2 and again at Time 3.

A Teacher report of Reactive and Proactive Aggression [50] was administered at Time 2 and Time 3.

### Parenting practices

The PDR (described above) can also be used to assess parenting practices. Parents were asked about the frequency of smacking over the previous 24 hours during the daily phone call at Time 2 and Time 3, as an index of harsh parenting.

### Main carer mental health

The twenty-eight item General Health Questionnaire (GHQ-28) [65] was administered at Time 1, Time 2 and Time 3 to provide an overall measure of carer mental health based on scores totalled across 4 domains: somatic symptoms, anxiety and insomnia, social dysfunction, and depression

### Demographic information

We recorded main carer and child ages, child gender (1 = male, 0 = female), family status (1 = single, 0 = two-parents), age the carer left full time education in school college or university (0 = < age 16 years, 1 = 17 + years), eligibility for free school meals (0 = no, 1 = yes), council/housing association home (0 = no, 1 = yes), car ownership (0 = no, 1 = yes) and ethnicity (1 = White British, 0 = all other ethnicities) at baseline. The index of multiple deprivation, based on postcode was calculated for each household [66]. A binary variable reflecting 1 = living in the most deprived quintile and 0 = all other quintiles was used.

### Interventions

Phase 1 intervention was the IY videotape parent management training (PMT) programme [20] delivered over 12–14 weeks according to the manual and in accordance with NICE (2006) guidelines [67]. All PMT therapists had completed an accredited IY training course.

Phase 2 interventions comprised RICAP and CTAU. RICAP was developed to further meet the individual treatment needs of these families by Hermione Roff working within the academic group at the University of Liverpool. As outlined earlier, RICAP is a brief 14 session manualised therapy which involves two therapists working in parallel with parent(s)/carer(s) and child. It was designed for children between the ages of 4 and 10 years. Table 1 gives a summary of the aims and components within the intervention. See Roff [56] for a full description of the approach.

RICAP was delivered by experienced CAMHS therapists across eight patch teams who were newly trained in the approach and who had completed two supervised cases prior to working with families on the trial. Treatment integrity was ensured and monitored in a number of ways; (a) three days of training using a manualised description of the key components of the therapy and illustration with case examples, (b) fortnightly supervision during completion of practice cases and study cases by the originator of the therapy, (c) examination of fidelity to the therapeutic model through checks on the inclusion of five key components i) the child is asked to make the drawings prescribed for each session as outlined in Table 1, ii) the therapist talks with the child about their drawings being curious about the process of drawing and what is portrayed, iii) the therapist provides a reflective summary of in-session conversations, alongside the child’s drawings and shares this book with the child at the next session. This included checks that reflections focussed on broadening the child’s understanding of thoughts and feelings that might underpin their own and others behaviours, (iv) the parent joins with the child at the start and at the end of each session to discuss a good time and a difficult time during the past week and plan for the week ahead, and (v) the parent received a therapeutic letter summarising new understandings made during the fortnightly parent-therapist sessions. All of these elements of the intervention were followed in every case, with the child’s book and the structured therapeutic letter providing a direct record for review in supervision. However, fidelity was not checked in greater detail, for example through the use of adherence checklists or independent fidelity ratings. The intervention was typically delivered over a 16 week time period.

CTAU comprised treatments offered routinely in the clinical settings, including individual parent behaviour management interventions, school-based intervention, family therapy, child cognitive behavioural therapy, anger management training, child psychotherapy, problem-solving skills training, supportive advice and listening, psychopharmacological intervention for ADHD, or a combination of these elements. Of those randomized to CTAU, 42% of the children received a school-based intervention component through Behavioural Education Support Teams (BEST) as an additional component of their CTAU. Examples of school-based intervention included restorative justice, emotional literacy, educational psychology involvement and broadening supports with school-based activities. The majority of families received two or three co-occurring clinical interventions typically parent and child and/or school focussed (63%). The most common clinical components of CTAU were individualised parent behaviour management training, ADHD diagnosis and medication, and anger management training.

### Statistical analyses

Feasibility was assessed in terms of recruitment and retention in the study. Data were analysed using *Stata* [68]. Skewed variables were transformed used square root transformation. Paired t-tests were used to determine the effect of phase 1 treatment. Repeated-measures Analysis of Covariance (ANCOVA) was used to test for effect of treatment allocation during phase 2 on post-intervention scores, after covarying for the possible effects of baseline child behaviour, child age, sex and deprivation [69]. Effect sizes were calculated as Cohen’s d, and statistical significance reported in relation to the p = .05 threshold. Intention to treat analyses are reported, assuming no change in scores for those cases lost to time 3 follow-up but who gave valid time 2 data. We report analyses with all allocated cases with complete follow up data, regardless of how many intervention sessions they received. Clinically reliable change using the borderline and clinical cut offs on the CBCL and SDQ is reported.

### Assignment

Randomisation occurred at the end of the time 2 assessment when outcome measures from phase 1 treatment were completed in the family home. If criteria for eligibility were met the next phase 2 individual treatment allocation on the schedule was then revealed.

## Results

### Recruitment and retention, feasibility

Recruitment and retention rates were used to assess feasibility. The CONSORT flow diagram for recruitment and retention to Phase 1 treatment is shown in Fig 1. It shows that a high proportion of parents (102/105; 97.1%) and teachers (94/102; 92.2%) completed measures before and after IY treatment.

Fig 2 shows the CONSORT diagram from assessment for eligibility to the Phase 2 RCT, recruitment and retention in the Phase 2 RCT.

There was a total of 72 among 102 (72.5%) children above the clinical threshold after parent training, 70 of whom did not have moderate to severe learning disability and so were eligible for phase 2. One family allocated to RICAP received CTAU after requesting an ADHD assessment, and no family allocated CTAU received RICAP. Completion rates in each arm of the trial were high and similar (28/31, 90.3%, for RICAP and 30/31, 96.8%, for CTAU).

The demographic characteristics of the sample recruited to Phase 1 are shown in Table 2. It is evident from the table that the families in the study were experiencing high levels of social and/or economic deprivation. Just under 70% of the sample were living in conditions equivalent to the lowest quintile for the UK population as a whole, using the Indices of Multiple Deprivation [66]. Over two-fifths of families reported being single parent households, and over two-thirds of the parents had low levels of parental education, having left school before the age of 16. Just under two thirds of the sample were eligible for free school meals. Only one family was not white British, reflecting the low level of ethnic minorities in the areas of Liverpool and the Wirral. Randomisation yielded two groups of families for Phase 2 intervention with similar profiles. The two groups did not differ in child age (*p* > 0.05) or the percentage of PMT sessions attended in Phase 1 (*p* > 0.05). They were also comparable in demographic composition and level of child antisocial behaviour symptoms on which randomisation was based (all p > 0.10).

**Table 2:** Characteristics of children and their families referred for parent management training for antisocial behaviour (Phase 1 treatment); whole sample and subgroups defined by status at start of phase 2 treatment.

	Phase 1	Baseline characteristics for Phase 2 subgroups				UK rate
	Whole sample N = 102	Non-clinical after Phase 1 treatment N = 28	Not randomised N = 12	Randomised to RICAP N = 31	Randomised to TAU N = 31	
Child male, n (%)	67 (65.7)	14 (50.0)	8 (66.7)	24 (77.4)	21 (67.7)	–
Child mean age in months (SD)	90.16 (22.0)	86.00 (21.4)	90.58 (22.9)	90.19 (22.0)	93.71 (22.5)	–
Main carer age in years (SD)	35.4 (7.35)	33.2 (5.78)	44.2 (9.37)	33.4 (6.89)	36.0 (5.87)	–
Mother left education by 16 years	70 (68.5%)	19 (67.8%)	9 (75.0%)	24 (77.4%)	18 (58.1%)	13%
Lone parent family	44 (43.1%)	11 (39.3%)	6 (50.0%)	13 (41.9%)	14 (45.2%)	7%
Most deprived UK IMD quintile	71 (69.6%)	20 (71.4%)	9 (75.0%)	22 (71.0%)	20 (64.5%)	20%
Eligible for free school meals	65 (63.7%)	16 (57.1%)	11 (91.7%)	22 (71.0%)	16 (51.6%)	18%
No car	46 (45.1%)	12 (38.7%)	7 (58.3%)	14 (45.2%)	12 (38.7%)	28%
Ethnicity white British	101 (99.0%)	30 (96.8%)	12 (100.0%)	31 (100.0%)	30 (96.8%)	91%
Council/housing association home	49 (48.1%)	9 (32.2%)	8 (66.7%)	16 (51.6%)	16 (51.6%)	17%

ǂ Data from *Social Trends* London: Office of National Statistics, 2000

### Phase 1, Parent Management Training Outcomes

Mean attendance for the PMT groups in Phase 1 was 7.41 (SD 4.6) sessions. There was a substantial decrease in parent-reported CBCL externalising (*d* = 0.73) and SDQ conduct problems (*d* = 0.68) and a small decrease on teacher-report TRF externalising (*d* = 0.27) and SDQ conduct problems (*d* = 0.22) from Time 1 to Time 2. The size of the treatment effects derived from the two parent outcome measures were similar in magnitude to those reported by Scott et al. (2001) in a UK community RCT comparing the effectiveness of PMT with wait-list controls in clinical settings. Table S2 in S2 File gives the means and standard deviations pre-and post-PMT intervention for the whole sample, and also subgroups defined by their status at the point of random allocation to phase 2 intervention.

### Phase 2, adherence to intervention

**Fig 3:**
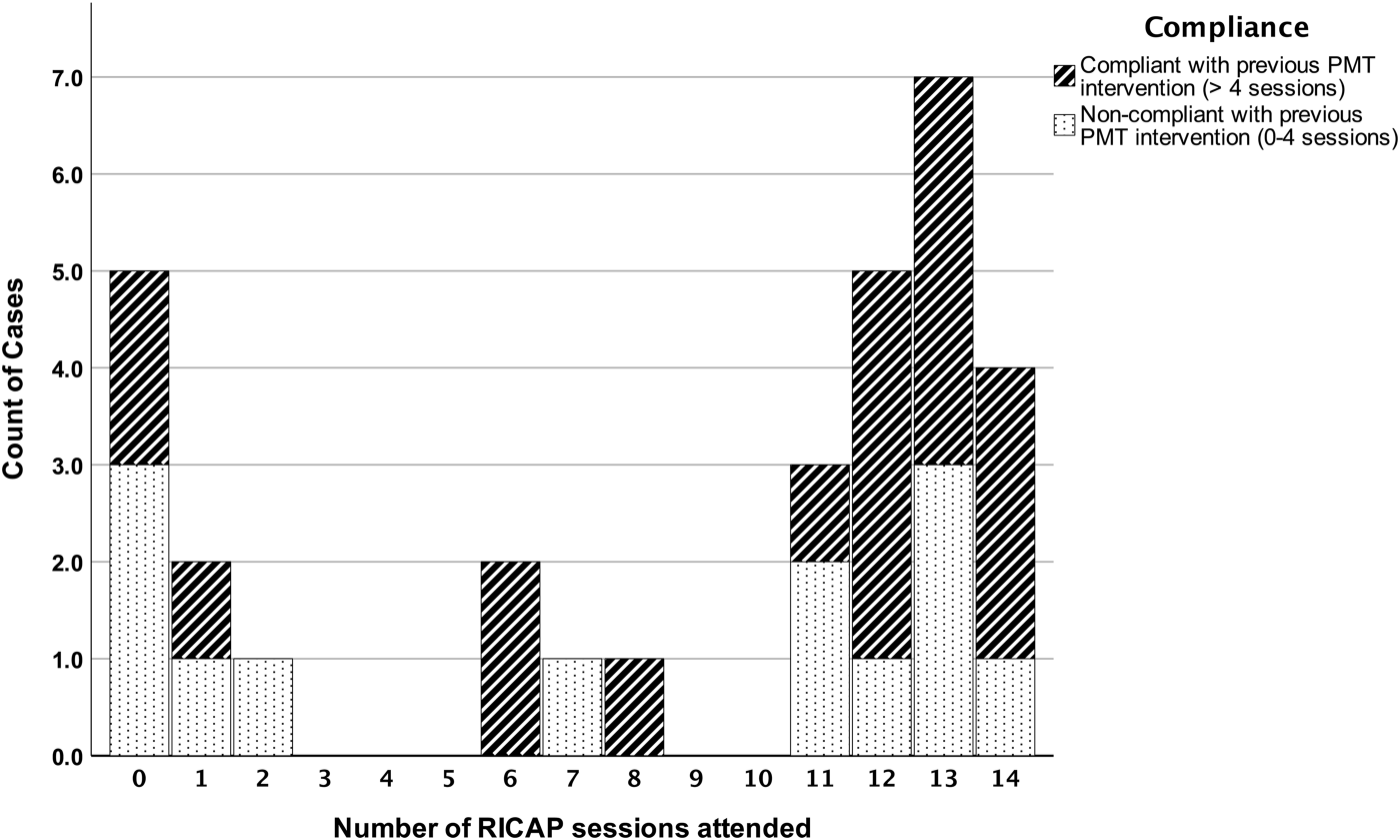
Participation in RICAP comparing families who attended five or more IY groups and those who attended no more than four.

Fig 3 shows the number of sessions of RICAP attended by families expressed as a function of the level of parent’s attendance during phase 1 PMT intervention. Since parent and child sessions occur on the same occasion, the maximum is 14 sessions together. Of the 31 who were randomised to receive RICAP, 23 (74.2%) attended for six sessions or more, deemed to be a therapeutic dose and 5 (16.1%) did not attend any sessions of RICAP. Importantly there were 13 families in the RICAP group where parents had attended four or fewer IY PMT groups (defined in Scott et al [3]) as drop-out), and of these seven attended eleven or more RICAP sessions. Within the CTAU arm 4/31 (12.9%) families did not attend any CTAU sessions. Adherence to CTAU could not be further determined as interventions varied and clinicians did not all define a set length of treatment at the start. Of note is the fact that 13/31 (41.9%) of CTAU recipients had an intervention that included a focus in both home and school settings, whereas RICAP recipients did not receive any school-based intervention.

### Phase 2 pilot RCT, child behaviour outcomes by parent report

The mean parent report scores at time 2 and time 3 with associated effect sizes are shown in Table 3 and Table 4. Analyses revealed significant small to medium treatment benefits associated with allocation to RICAP intervention on externalising problems (*d* = .37), internalising problems (*d* = .46) and on total child problems reported on the CBCL (*d* = .43). These benefits were reduced by 9–16% in intention to treat analyses but remained small to moderate and significant. These effects were not seen with parent-report SDQ nor Parent Daily Report measures.

**Table 3:** Summary of parent raw report scores before and after phase 2 intervention with per protocol and intention to treat effect sizes based on adjusted scores.

Parent report	RICAP Therapy Mean (SD)			Treatment as Usual Mean (SD)			Differences between phase 2 intervention groups			Intention to treat analysis (TAU N = 31; RICAP N = 31)		
	N	Before Time 2	After Time 3	N	Before Time 2	After Time 3	Difference in mean* (95% CI)	Partial eta squared; P value	Cohen’s d	Difference in mean* (95% CI)	Partial eta squared P value	Cohen’s d
CBCL Externalising	28	29.89 (11.23)	22.93 (11.04)	30	28.80 (11.81)	28.07 (11.10)	6.00 (1.84, 10.17)	.139; 0.006	.37	5.07 (1.01, 9.12)	.101; 0.015	.32
CBCL Internalising	28	14.25 (9.68)	10.29 (7.03)	30	12.90 (6.86)	16.60 (10.72)	7.36 (3.39, 11.33)	.210; 0.001	.46	6.77 (2.93, 10.6)	.180; 0.001	.42
CBCL Total problems	28	70.39 (27.71)	53.61 (25.28)	30	65.67 (27.55)	70.37 (32.02)	20.20 (8.23, 32.17)	.181; 0.001	.43	17.83 (6.22, 29.4)	.145; 0.003	.38
SDQ Total problems	28	19.93 (5.72)	18.75 (6.23)	30	19.00 (6.26)	18.23 (6.26)	0.019 (−2.61, 2.65)	.000; 0.989	.00	−0.44 (−2.99, 2.13)	.002; 0.730	.05
SDQ Conduct problems	28	5.52 (2.20)	4.93 (1.92)	30	5.63 (2.39)	5.37 (2.22)	0.297 (−.50, 1.09)	011; 0.459	.10	0.13 (−.67, 0.93)	.002; 0.740	.05
PDR Total problems/day	20	12.47 (4.94)	10.48 (4.69)	25	11.06 (4.79)	9.69 (5.45)	−0.16 (−2.44, 2.13)	.000; 0.891	.00	0.49 (−2.45, 1.46)	.005; 0.615	.07
PDR Use of smacking	24	0.072 (0.09)	0.024 (0.05)	27	0.097 (0.21)	0.074 (0.20)	0.05 (−0.04, −0.14)	.028; 0.262	.17	.021 (−0.06, 0.10)	.006; 0.574	.08
GHQ 28	28	7.71 (8.52)	5.00 (6.15)	30	8.93 (11.24)	6.83 (8.66)	1.25 (−2.34, 3.84)	.009; 0.489	.09	−0.16 (−3.99, 3.67)	.000; 0.930	.00

CBCL = Child Behaviour Checklist, SDQ = Strengths & Difficulties Questionnaire, PDR = Parent Daily Report, GHQ = General Health Questionnaire *Difference in time 3 scores between treatment arms, adjusted by analysis of covariance for initial score (time 2), child age, sex and deprivation. Intention to treat analysis based on 29 TAU and 30 RICAP cases with teacher informant data at time 2, 3 children were not attending school.

**Table 4:** Summary of teacher raw report scores before and after phase 2 intervention with per protocol and intention to treat effect sizes based on adjusted scores.

Teacher report		RICAP Therapy Mean (SD)			Treatment as Usual Mean (SD)			Differences between phase 2 intervention groups			Intention to treat analysis¥ (TAU N = 29; RICAP N = 30)		
	N		Before Time 2	After Time 3	N	Before Time 2	After Time 3	Difference in mean* (95% CI)	Partial eta squared; P value	Cohen’s d	Difference in mean* (95% CI)	Partial eta squared; P value	Cohen’s d
TRF Externalising	27		19.07 (16.95)	16.81 (12.66)	28	19.50 (14.39)	16.46 (15.09)	−0.68 (−6.63, 5.27)	.001; 0.820	.03	−0.22 (−5.78, 5.35)	.006; 0.938	.08
TRF Internalising	27		6.44 (7.02)	10.00 (8.42)	28	7.86 (6.40)	8.14 (8.23)	−2.58 (−7.20, 2.05)	.025; 0.268	.16	−1.74 (−6.08, 2.59)	.012; 0.423	.11
TRF Total problems	27		45.67 (32.93)	47.74 (27.67)	28	45.00 (31.93)	42.86 (36.33)	5.01 (−19.75, 9.73)	.009; 0.498	.09	−3.31 (−17.04, 10.43)	.004; 0.631	.06
SDQ Total problems	27		12.81 (7.76)	13.70 (7.05)	28	12.89 (6.61)	10.39 (6.56)	3.57 (−6.68, −0.67)	.111; 0.017	.33	−3.09 (−5.83,-0.36)	.089; 0.027	.30
SDQ Conduct problems	27		2.85 (2.83)	2.30 (2.13)	28	3.14 (2.66)	2.04 (2.13)	−0.38 (−1.42, 0.66)	.011; 0.467	.10	−0.29 (−1.26, 0.67)	.007; 0.547	.08
Proactive aggression	27		5.56 (2.99)	5.26 (2.77)	27	5.67 (3.00)	4.85 (2.30)	−.54 (−1.89,.813)	.013; 0.427	.11	−0.28 (−1.55, 1.00)	.004; 0.662	.06
Reactive aggression	27		9.15 (3.44)	9.31 (3.97)	26	10.15 (3.67)	7.81 (2.94)	−1.84 (−3.46, −0.22)	.100; 0.027	.32	−1.57 (−3.16, 0.01)	.072; 0.052	.27

TRF = Teacher Report Form, SDQ = Strengths & Difficulties Questionnaire *Difference in time 3 scores between treatment arms, adjusted by analysis of covariance for initial score (time 2), child age, sex and deprivation. Intention to treat analysis based on 29 TAU and 30 RICAP cases with teacher informant data at time 2, 3 children were not attending school.

*Clinically reliable change –* Table S3 in S2 File shows the percentage of participants rated within the normal, borderline or clinical ranges on the parent-report CBCL and SDQ. The proportion of children rated in the clinical range for CBCL externalising fell by 30.0% in the RICAP arm, and by only 4.1% in the CTAU arm, leaving 57.1% and 75.9% still in the clinical range at the end of intervention respectively. The proportion rated in the clinical range for internalising problems reduced by 27.0% in the RICAP arm, and increased by 15.0% in the CTAU arm, leaving 21.4% and 51.7% still in the clinical range at the end of intervention respectively. Clinically reliable change according to the SDQ measure did not differ between treatment arms.

### Phase 2, Outcomes from Teacher Reports

In contrast to parent reports, no group differences were observed in teacher rated reports in favour of RICAP. No group differences were observed on either the CBCL scales or the SDQ conduct problems subscale. Teachers reported lower levels of reactive aggression at time 3 in the CTAU group compared to the RICAP group. No differences were reported in proactive aggression. Teachers reported significantly lower levels of SDQ total problems at time 3 in the CTAU group. Intention to treat analysis revealed a similar pattern of results.

*Clinically reliable change* –The proportion of children rated in the clinical range within the school setting was observed to be lower than that reported in the home setting as a whole, but there were no differences between the RICAP and CTAU groups.

## Discussion

This pragmatic RCT confirmed previous evidence that many children do not improve with parent training. We demonstrated that we could retain these families in a second intervention, including many who dropped out of parent-training, and follow them up over a substantial period of time, with equal numbers retained in both arms of the intervention. We showed that UK NHS practitioners can be trained and supervised in this novel therapeutic approach. RICAP was associated with more favourable clinical outcomes than CTAU for parent-reported problems on the CBCL in children who had not responded to parent-training. However, this was not the case for some teacher reported problems where there was evidence for more favourable outcomes following the CTAU intervention.

Feasibility might be a concern for studies of conduct problems in children, because these are associated with socioeconomic deprivation [10], which in turn is associated with lower participation in medical research [70]. However the findings provide strong support for the feasibility of conducting a two-phase trial of the treatment of conduct problems within the UK NHS. Notably there was a high level of retention of families over a period of at least a year, and a high completion rate of measures from parents and teachers. Of those recruited to the first, parent training, phase of the study, 97% provided follow up data, and of these, 89% consented to be randomized. 94% of those randomised provided follow up data. There was no evidence of differential attrition between the RICAP and CTAU groups.

As far as we are aware this is the first evaluation of an intervention designed to treat conduct problems which have not responded to ‘gold-standard’ parent-training. In the first phase of the study, parent-training was associated with moderate to large reductions in parent-reported conduct problems. Despite this reduction, 72.5% of children (72/102) remained in the clinical range according to parent or teacher report after parent-training and 70/72 were eligible for phase 2. Allocation to RICAP was associated with significant small-to-moderate reductions from post-phase 1 to post-phase 2 in parent-reported CBCL externalising, internalising and total problems. Allocation to RICAP was not associated with a significant reduction for parent-reported SDQ or daily report of problems compared to CTAU, and no significant treatment effects were observed for teacher ratings. The results provide preliminary evidence that RICAP delivered in regular clinical practice may be an effective and acceptable intervention for treatment-resistant conduct problems. This is encouraging when viewed in the context of the majority of studies of treatment for child conduct problems which only include parent reported outcomes. Teacher reported outcomes in this study provided a different picture, albeit on different measures. These indicated superiority of CAMHS based CTAU interventions.

Interpretation of the effect sizes from this pilot trial should be considered in the context of several contextual factors. First, the sample represented complex cases who have not responded to parent-training. These families were characterised by high rates of risks for conduct problems including low parental education and deprivation. A greater proportion of the sample remained in the clinical range after parent-training compared to prior UK RCTs [3]. Second, this study was conducted with an unselected sample of referred children in usual clinical services and so is likely to be representative of children attending clinical services. Third, the trial was conducted by CAMHS practitioners after brief training in RICAP. They had typically only experienced being supervised with two practice cases prior to the trial proper. Therefore, the effect sizes observed in this pragmatic trial may underestimate what might be achieved with more experienced RICAP therapists within clinical practice over time. In this context the small to moderate treatment gains in children allocated to RICAP provide promising preliminary indications of potential benefit for treatment-resistant conduct problems, which require examination in an adequately powered trial. They were comparable to those reported in a meta-analysis of 52 evidence-based psychosocial interventions which were tested against usual clinical care [71].

UK NICE (2013) [4] guidelines acknowledge that complex families may need alternative approaches to first-line group-based parenting interventions devised to better meet their needs. However, rather than offer alternative approaches as a first line of approach, offering them once the outcomes from parent training is known, ensures both that everyone has had an evidence based intervention, and that they are being targeted to those most in need. The method and process of RICAP was devised specifically with the complexities of these families in mind. Whilst providing a structure for the therapists to work within, the considerable flexibility of formulation within the RICAP method allows different aspects of child and/or parent vulnerability to be addressed, arguably making it more sensitive to the heterogeneity evident in the most complex families. A substantial proportion of those families who did not adhere to the PMT intervention subsequently successfully adhered to RICAP (62%), indicating that the intervention was acceptable to these families. Unfortunately, it was not possible to examine comparable adherence to CTAU.

It is noteworthy that RICAP was associated with similar magnitude differences in internalising and externalising problems. Recent meta-analyses of the effects of parent-training on internalising outcomes have concluded that there is evidence for a small effect but with significant heterogeneity across studies [72,73]. The focus in RICAP on children and parents’ awareness of thoughts and feelings and the targeting of anxieties which may underlie oppositional behaviour may mean that it is particularly suited to reducing co-occurring internalising problems. While differences favouring RICAP were observed for parent-reported CBCL externalising and internalising outcomes, there were no differences on parent-reported SDQ scores, on behaviours reported by parents by phone in the PDR, or teacher reported CBCL scores.

The lack of group differences on parent and teacher SDQ conduct problems scores may reflect that it was designed as an epidemiological screening tool, and its small number of items may make it less sensitive to change than the CBCL. In addition, two out of five conduct problem items (lying and stealing) are less relevant for younger children. Smaller effect sizes for the SDQ compared to the CBCL or the Eyberg Child Behaviour Inventory, another lengthier measure, have been found in previous RCTs [3,74].

The inclusion of teacher as well as parent reported behaviours is found in only a minority of trials of parent training [75]. This yielded findings that require further examination for three main reasons. First interpretation of the contrasting outcomes for parent and teacher rated behaviours is not straightforward because they were not apparent on the same measures. Parent reported behaviours on the CBCL externalising, internalising and total problems, but not SDQ conduct problems showed an advantage for RICAP, while teacher reported behaviours showed an advantage for SDQ total, but not conduct problems, and not any of the CBCL scales.

Second, while it is not possible to infer from our findings with this modest sample size that RICAP did not lead to comparable effects on teacher rated outcomes, it is possible that RICAP has an effect which is confined to behaviours shown within the family. In particular, RICAP is an intervention focussed on improving the parent-child relationship, addressing attachment related concerns and enhancing reflection on the meaning of behaviours in the context of parent-child interactions. As the focus is on creating change in the home setting this may not generalise to the school setting.

Third, there were, as summarised above, some indications that the CTAU group showed greater improvement than the RICAP group on teacher rated problems. This may reflect that randomization generated a second contrast. The study took place at a time when routine CAMHS care sometimes included school-based interventions. These included emotional literacy, educational psychology involvement and broadening supports with school-based activities. Children in the RICAP arm by contrast, did not receive any school-based interventions. The preliminary evidence of an advantage on some measures for those who received a school-based intervention, suggests that RICAP should be expanded to include a ‘relationships in school’ element aiming to increase reflective functioning in that setting ideally including sessions with teacher and child. A school-based version of the Incredible Years programme has been developed and shown to be associated with a small effect on teacher-reported outcomes [76]).

Strengths of the study include that it was a pragmatic trial [77], run within community settings, employing regular clinical staff, and it did not exclude cases on the basis of comorbidity. These design elements enhance the generalisability of the findings from the study. The extent to which trial outcomes reported are ecologically valid is important [78] and RCTs are hard to complete in real-life settings. One possible source of bias in pragmatic trials can be clinician de-selection of vulnerable children. To address this the study used consecutive referrals to parenting groups which meant that this was very unlikely. The initial rate of consent to take part in the study was high and very few eligible families refused randomisation; one declined in order to choose child psychotherapy and 3 families did not want further intervention after PMT. One child was admitted to an inpatient unit by his clinician.

In spite of the advantages of the CTAU arm for generalizability, outlined above, and the serendipitous introduction of school-based intervention in one, but not the other arm, the use of the local CTAU also introduced another limitation. The extent of the different interventions offered to children, in the family setting and/or the school setting, introduced a heterogeneity that was not documented or controlled in the study, and this could affect generalizability. Heterogeneity in usual care in pragmatic clinical trials is a widely accepted limitation of the test of real-world effectiveness [79].

A further major limitation of the study was associated with parent report. Parents were not blinded to treatment, and parent report may have simply reflected greater satisfaction among parents for RICAP than for CTAU. Teachers by contrast had not received either intervention and were blind to the intervention status of the child. A further limitation is that both CTAU and RICAP followed PMT and it is therefore not clear whether their effectiveness was potentiated by this first intervention. Interpretation of the PDR findings is limited by smaller numbers of parents completing this measure than the others, 20/28 in the RICAP group and 25/30 in CTAU which could have introduced unknown biases in the subsample providing PDR data. The smaller number of parents reporting suggests that this measure is less feasible than others for use in trials. We have been unable to locate any RCTs which used both the CBCL and the PDR, so it remains unclear whether it is a valid measure of treatment effect. The sample was overwhelmingly white British so the findings may not generalise to other ethnic groups. Registration of the trial after data collection had started was a limitation. However, the study protocol which was submitted to the Liverpool Children’s and Wirral NHS ethical committees prior to the start of recruitment remained unchanged from that point.

Although the first phase of the study was uncontrolled, so direct comparison with published trials cannot be made, there were indications that numbers still in the clinical range following IY were higher than in other studies [3]. We suggest three possible explanations. First our children were considered to be in the clinical range following IY if they were above threshold either on the CBCL or SDQ, either by parent or teacher report. In view of the importance of conduct problems outside of the family to peer and educational development we regard this as a strength, which also likely lowered the threshold for selection into the trial. Second, again in contrast to Scott [3], adherence to IY was not monitored which may have affected the effectiveness of the intervention, and third there was a higher drop-out from IY, of 40% in this study compared to 19% in the study of Scott [3].

In conclusion, this study demonstrated the feasibility of a design to evaluate a new intervention for the clearest target that we know of for early intervention to improve the mental health of children, adolescents and adults. It introduces a key step towards personalisation based on lack of responsiveness to a first line treatment for the disorder [80]. The new RICAP intervention was highly acceptable to parents and children, and addresses a plea made by the parents of children who are offered parent training, that assessments and treatments should focus as much on the child as on the parents [33].

Based on the findings there is a need for a full-scale RCT of RICAP, with sufficient statistical power to examine predictors of outcomes. For example, our previous work has indicated that maternal unresolved attachment status, which is associated with lower reflective capacity, is associated with poorer outcome for IY parent management training [81]. It may be that this would also be the case for RICAP because of its emphasis on enhancing parental reflection about their child’s thoughts, feelings and behaviour in order to guide their own choice of parental response. In turn this may point to the future need for modifications of RICAP to address parental attachment processes.

A full-scale RCT would also permit examination of whether a modification of RICAP to explicitly include interpersonal behaviour at school would improve teacher-rated outcomes. Many of the ideas and techniques in RICAP, which currently focus on the child’s behaviour within the family context and the parent-child relationship, are well suited to adaptations to a focus on the child and their peers, and the child in the classroom, and hence potentially to an approach tailored to the particular social contexts of the child’s behaviours. The development of such an approach would likely require a phase of co-production with parents and teachers in order ensure a fit with their complementary roles and the contrasting demands of different social domains [82], and a degree of personalization in order to match the approach to the needs of the child [33].

## Supporting Information

S1 File Trial protocol. (DOCX)

S2 File The sample size calculation, information provided to parents, the trial checklist, and additional tables S1 and S2 are shown in the Supplementary Materials. (DOCX)
