# Supplementary material for: ‘Pilot RCT of a new treatment for child conduct problems that have not responded to evidence-based parent training’
Source: PLoS One. 2026 Jul 28;21(7):e0353611. doi: 10.1371/journal.pone.0353611 (PMC13411892; doi:10.1371/journal.pone.0353611)
Supplement: S1 File — (DOCX) [file pone.0353611.s001.docx]

| **Title**: **Matching the therapeutic approach to help to children’s emotional and behavioural needs: a comparison of different ways of helping.**  **Pertinent Background**: There is overwhelming evidence that conduct problems evident in the preschool and primary school years are strongly predictive of antisocial personality disorder in adult life, as evidenced in antisocial behaviours and associated social dysfunction (Moffitt et al 2002). The poor outcomes extend well beyond, to DSM Axis I disorders notably depression and drug and alcohol abuse, and to partner violence, negative parenting, and physical abuse, and hence to risk to the next generation. Although the prevalence of conduct problems in girls is lower, there do not appear to be gender differences in their causes and the consequences.  There is therefore strong justification for early intervention for conduct problems. Much of the research has focussed on various forms of parent management training, which are derived from social learning based theories of children’s antisocial behaviours. These seek to increase positive interactions between parents and children, to reduce inadvertent reinforcement of disruptive behaviours, and to establish consistent and effective methods for dealing with noncompliance. There is consistent evidence for their effectiveness (Kazdin 2001), although until recently there had been few studies outside of university and similar specialist US treatment settings (Scott et al 2001). Scott and colleagues showed, using a waiting list control design, that a group based approach devised by Carolyn Webster Stratton (Webster-Stratton et al 1998), was effective with a referred inner-city and suburban sample of children aged 3-8.  Although recent progress in the area has been encouraging, the interventions that are available have clear limitations. Firstly a substantial minority of children are not helped by current treatments for conduct problems. This arises because some parents do not take up parent training, or drop out, and there are children who do not respond to their parents’ improved skills. The estimate of the size of the drop out and treatment failure groups depends to a certain extent on the definitions used, and is likely to vary depending on sample characteristics. For example Kazdin et al (1994) found that 40% dropped out, and Scott et al (2001) that one third were still diagnosable with ODD following treatment. Attempts to improve outcomes have been made by broadening the scope of the work with parents and by adding therapeutic work with children. A major concern is that the poor response is generally found to be associated with factors that also are more likely to be associated with more persistent forms of disorder, including more severe and chronic conduct problems (Kazdin et al 1994), marital discord, harsh parenting practices and unresolved loss or trauma in parents (Routh et al 1995, Kazdin 2001). There is a need therefore to develop treatment approaches that are effective with poor responders to parent training. Secondly it is evident that, although maladaptive patterns of reinforcement probably have a role in generating and maintaining conduct problems, individual vulnerabilities make a major contribution. These include limited verbal skills, deficits in executive function thought to be associated with prefrontal cortex dysfunction, information processing biases contributing to misperception of hostile cues from others and to inflated appraisal of competence, insecure attachment and limitations in interpreting and responding to other people’s emotions (Hill 2002). Very little is known about the way these contrasting vulnerabilities interact, nor how they operate over time, however the research underlines the extent of the individual difficulties of many children with conduct problems, which suggests that a major aim of new treatments should be to help children overcome them  **Research Question:**  Is a randomized controlled trial of RICAP with hard to treat children with conduct problems feasible?  **Objectives and Specific Aims:**  The main aims are to establish the recruitment and attrition rate to a study with this design, to test whether the randomisation process is acceptable, to examine for differences in completion rates in the two arms of the randomised study, to compare a range of outcome measures, and to generate data for a power calculation to provide the basis for an application to carry out a full scale randomised controlled trial.  **Design:**  We will carry out a pilot randomised controlled study of a novel treatment for conduct problems that have not responded to parent training. It is designed according to the MRC guidelines on Phase Two Exploratory Studies of complex interventions (MRC 2000) and the CONSORT statement (Altman, 1996: Moher, Schulz, Altman, Lepage & for the CONSORT Group, 2001)  **Basic Demographic Data:**  A total of 160 children and their parents will be recruited to the study, and will be assessed after 4 months and 1 year. Of the initial sample of 160 we expect 140 to attend for the second assessment, 120 for the one year follow up. The criterion for inclusion in the study will be a score in the clinical range on either the CBCL/TRF externalising subscale (Achenbach 1991 a, b) or SDQ conduct problems subscale from parent and/or teacher informant (Goodman 1997) and they are aged 5 – 10 years. They will be assigned to a parent training programme, and will be reassessed after 4 months (Phase 1). The 70 with the poorest outcomes will be randomised to further clinical treatment as usual or to the new treatment – The Reflective Interpersonal Therapy for Children and Parents (RICAP) (Phase 2). Poor outcome will be defined as ratings in the clinical range either on parent or teacher reported CBCL, TRF or SDQ. Within the resource constraints of the study it will not be possible to generate the pilot intervention sample from an initial sample of greater than 160, so if the eligibility rate to enter the randomization falls below 50% the threshold will be altered. All parents, including those who do not start either treatment, those who drop out, and those who complete will be followed up.  **Methods**  *Demographic Information*  Sex and age of child, family composition, parents’ education and current employment, post code.  *The Strengths and Difficulties Questionnaire (Goodman 1997)*  This is a widely used well validated brief measure of emotional and behavioural problems (Goodman 1997). Parent and teacher versions will be used.  *Child Behavior Checklist (CBCL)* (Achenbach 1991a), Teacher Report Form (Achenbach 1991b)  Extensively used questionnaire measure of a wide range of externalising and internalising behaviours.  *The Parent Daily Report (Chamberlain and Reid 1987)*  This records 36 behaviours as present or absent each day for week, and has been widely used as an alternative to direct observations in the home.  *Teacher Report of Reactive/Proactive Aggression (Dodge and Coie 1987*)  This is a widely used 6-item teacher questionnaire which has excellent psychometric properties.  *The General Health Questionnaire GHQ-28 (Goldberg and Hillier 1979)*  This is a widely used, reliable and valid brief measure of current anxiety and depression. GHQ-28 scores predict psychiatric disorder identified by standardised interview (Goldberg et al 1997), and the depression sub-scale shows moderately good agreement with current DSM depression Koeter (1992).  **Methods**  The procedure for explaining the study to children and parents is described under H, ‘Procedures’.  The interventions are described here:  Parent-Training  The parent training package is well established and has been described in numerous publications (e.g. Webster-Stratton et al 1998, Scott et al 2001). It has an explicit structure described in a manual. The applicant arranged for Dr Webster-Stratton to come to Liverpool in 1993, to train a group of 10 mental health professionals, at least 4 of whom will be involved with this project. Since then the approach has become widely used in Liverpool and on the Wirral, and Hermione Roff a research therapist working with the applicant has provided training to other groups. Each group lasts for between 12 and 14 weeks.  Intensive Child and Parent Treatment  The Reflective Interpersonal Therapy for Children and Parents has been devised by Hermione Roff working with the applicant. RICAP is a fourteen week structured treatment for children and their parents. Two therapists and the parent and child all meet together once at the start of therapy (set-up meeting). The child is then seen by one therapist for twelve weekly sessions, and the core activity is the creation and review of a notebook of child’s drawings and a record of conversations between the child and the therapist. Conversation, based on the drawings and the child’s commentary on them, focuses on the explanation of behaviours in terms of the child’s emotions and beliefs about his/herself, and others in the family and the wider social world. There is a planned sequence of topics dealing with interpersonal understanding and behaviour. At the beginning of each of these twelve sessions the parent joins for the first ten minutes during which she/he is asked to tell the child’s therapist of a good and a bad event that has occurred during the previous week. The parent also joins for the last ten minutes of these sessions. During this period the parent receives six separate fortnightly sessions from the second therapist focusing on their understanding of their child’s thoughts, feelings, and behaviours, and identifying accurately positive and negative behaviours. Finally, parent, child and therapists meet once at the end of therapy (review meeting).  Control Group  Children randomised to the control group will receive further clinical treatment which is likely to vary across child mental health teams, and will include further help with parenting skills, support for parents, family therapy and occasionally individual therapy for the child.  **Data analyses**  The distributions of scores for all measures will be examined, and log transformed where appropriate for parametric analyses. Where transformation fails to provide an acceptable distribution, results will be checked using non-parametric equivalents.  All analyses will be carried out on an intention to treat basis.  *Phase 1 (N = 140)*  Scores on each of the measures at baseline and at 4 months will be compared using two tailed paired t-tests.  *Phase 2 (N = 70)*  The main purpose of phase two is to carry out a pilot study to test the integrity of the trial protocol for the evaluation of the RICAP, and to provide estimates of means and standard deviations of the measures in ‘treatment as usual’ and RICAP groups. Exploratory analyses comparing the RICAP and control groups will be conducted using analysis of covariance, entering behavioural scores at randomisation as covariates, together with age and sex of child, and the Townsend index of deprivation based on post codes.  **Sample Size**  Browne (1995) recommends that in a pilot study 30 are needed in each group to provide reasonably narrow confidence intervals for the standard deviations required for a power calculation. The 80% upper one-sided confidence limit will be used when determining power for an RCT.  **Dissemination of Results**  Publication in peer review journals and presentation at meetings for the relevant professionals. Caution will need to be exercised in recommending RICAP even if this study suggests it is effective, prior to a full scale RCT.  **REFERENCES**  Achenbach, T.M. (1991a). *Manual for the Child Behaviour Checklist/4-18 and 1991 Profile.* Burlington, VT: University of Vermont, Department of Psychiatry.  Achenbach, T.M. (1991b). *Manual for the Teacher’s Report Form and 1991 Profile.* Burlington**,** VT: University of Vermont, Department of Psychiatry.  Altman, D. G. (1996). Better reporting of randomized controlled trials: the CONSORT guidance, *British Medical Journal*, 313, 570 – 571.  Browne RH. (1995). On the use of a pilot sample for sample size determination. *Statistics in Medicine, 14, 1933-1940.*  Chamberlain P and Reid JB. (1987). Parent observation and report of child symptoms. *Behavioural assessment, 9, 97-109.*  Dodge KA and Coie JD. (1997). Social information processing factors in reactive and proactive aggression in children’s peer groups. *Journal of Personality and Social Psychology, 53, 1146-1158.*  Goldberg D P, and Hillier V F (1979). A scaled version of the General Health Questionnaire. *Psychological Medicine,* 9, 1, 139 – 145.  Goldberg D P, Gater R, Sartorius N, Eustun T B, Piccinelli N and Gurege O and Rutter C. (1997). The validity of two versions of the GHQ in the WHO Study of mental illness in general health care. *Psychological Medicine,* 27, 191 - 197.  Goodman R. (1997). The Strengths and Difficulties Questionnaire: a research note. *Journal of Child Psychology and Psychiatry, 38, 581-586.*  Halperin JM, McKay KE and Newcorn JH. (2002). Development, reliability, and validity of the children’s aggression scale – parent version. *Journal of the American Academy of Child & Adolescent Psychiatry 41, 245-252.*  Hill J. (2002). Biological, psychological and social processes in the conduct disorders. *Journal of Child Psychology and Psychiatry, 43, 133 – 164.*  Kazdin AE. (2001). Treatment of conduct disorders. In Eds J Hill and B Maughan. *Conduct disorders in childhood and adolescence. Cambridge: Cambridge University Press.*  Kazdin AE, Mazurick JL and Siegel TC. (1994). Treatment outcome among children with externalising disorder who terminate prematurely versus those who complete psychotherapy. *Journal of the American Academy of Child & Adolescent Psychiatry 33, 549-557.*  Koeter, MWJ (1992). Validity of the GHQ and SCL Anxiety and Depression Scales: A comparative study*. Journal of Affective Disorders, 24, 271 - 280.*  MRC (2000) *A framework for development and evaluation of RCTs for complex interventions to improve health*.  Moffitt TE, Caspi A, Harrington H and Milne BJ. (2002). Males on the life-course persistent and adolescence – limited antisocial pathways: follow-up at 26 years. *Development and Psychopathology, 14, 179-207.*  Moher, D., Schulz, K.F., Altman, D.G., Lepage, I. & for the CONSORT Group (2001). The CONSORT statement: revised recommendations for improving the quality of reports of parallel-group randomised trials. *Lancet*, 357, 1191-1194.  Routh CP, Hill JW, Steele H, Elliott CE and Dewey ME. (1995). Maternal attachment status, psychosocial stresses and problem behaviours: follow-up after parenting courses for conduct disorder. *Journal of Child Psychology and Psychiatry, 36, 1179-1198.*  Scott S, Spender Q, Doolan M, Jacobs B and Aspland H. (2001). Multicentre control trial of parenting groups for childhood antisocial behaviour in clinical practice. *British Medical Journal, 323,*  Webster-Stratton C and Hancock L. (1998). Training for parents of young children with conduct problems: content, methods and therapeutic processes. In Briesmeisder JM, Schaefer CE, Ed. *Handbook of parent training.* 2^nd^ Ed New York: Wiley. |
| --- |
