# Supplementary material for: ‘Pilot RCT of a new treatment for child conduct problems that have not responded to evidence-based parent training’
Source: PLoS One. 2026 Jul 28;21(7):e0353611. doi: 10.1371/journal.pone.0353611 (PMC13411892; doi:10.1371/journal.pone.0353611)
Supplement: S2 File — (DOCX) [file pone.0353611.s002.docx]

**Supplementary Materials 1:** Calculation of sample size

The study design anticipated that 160 families would be needed in Phase 1 in order to generate a total of 60 for randomization at time 2. This was based on an attrition rate of 25% and a prediction that around 50% of the children would remain in the clinical range following parent training. The sample size for randomisation was based on Browne’s (1995) recommendation that in a pilot study 30 participants are needed in each group to provide reasonably narrow confidence intervals for the standard deviations required for a power calculation.

**Supplementary Materials 2:** Information given to participants

In order to minimise expectancy effects parents were told at the point of consent before phase 1 intervention that the study was designed to compare two different ways of working with children and parents after parent training. The information sheets were worded carefully. The study was entitled ‘Matching therapy to children’s needs: a comparison of different ways of helping’.

Parents were told:

***What is the study about?***

*Research shows that most children are helped when their parents participate in parenting groups like the one you have been invited to join. Some children also seem to benefit from having their own additional time with a therapist. However, research does not yet tell us which is the best way to give this additional help. That is what we want to find out. The parenting group you have been invited to join lasts for between 12 and 14 weeks. If after you have attended the parenting group, you, or your child’s teacher, tell us in a questionnaire that your child is showing behaviour problems above a certain level, you will then be offered one of two possible types of further help.*

***Why do we need to compare two types of help?***

*We want and need to know which is the better way of working with children. In one method, therapists have a set step-by-step plan and work through this with you and your child. In the other, the therapist works through the difficulties you and your child want to talk about each time you meet. Both of the methods have been used before and are still being used today to help children. But we still do not know which is the better type of help. The results of this research study should hopefully give us an answer.*

**Table S1**

**
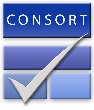
CONSORT 2010 checklist of information to include when reporting a pilot or feasibility trial***

| **Section/Topic** | **Item No** | **Checklist item** | **Reported on page No** |
| --- | --- | --- | --- |
| **Title and abstract** | | | |
|  | **1a** | **Identification as a pilot or feasibility randomised trial in the title** | **1** |
|  | **1b** | **Structured summary of pilot trial design, methods, results, and conclusions (for specific guidance see CONSORT abstract extension for pilot trials)** | **2 - 3** |
| **Introduction** | | | |
| **Background and objectives** | **2a** | **Scientific background and explanation of rationale for future definitive trial, and reasons for randomised pilot trial** | **4-15** |
|  | **2b** | **Specific objectives or research questions for pilot trial** | **15** |
| **Methods** | | | |
| **Trial design** | **3a** | **Description of pilot trial design (such as parallel, factorial) including allocation ratio** | **16 - 17** |
|  | **3b** | **Important changes to methods after pilot trial commencement (such as eligibility criteria), with reasons** | **n/a** |
| **Participants** | **4a** | **Eligibility criteria for participants** | **16** |
|  | **4b** | **Settings and locations where the data were collected** | **15-16** |
|  | **4c** | **How participants were identified and consented** | **15 - 16** |
| **Interventions** | **5** | **The interventions for each group with sufficient details to allow replication, including how and when they were actually administered** | **8 - 14** |
| **Outcomes** | **6a** | **Completely defined prespecified assessments or measurements to address each pilot trial objective specified in 2b, including how and when they were assessed** | **17 - 19** |
|  | **6b** | **Any changes to pilot trial assessments or measurements after the pilot trial commenced, with reasons** | **n/a** |
|  | **6c** | **If applicable, prespecified criteria used to judge whether, or how, to proceed with future definitive trial** | **n/a** |
| **Sample size** | **7a** | **Rationale for numbers in the pilot trial** | **Supplement** |
|  | **7b** | **When applicable, explanation of any interim analyses and stopping guidelines** | **n/a** |
| **Randomisation:** |  |  |  |
| **Sequence**  **generation** | **8a** | **Method used to generate the random allocation sequence** | **16** |
|  | **8b** | **Type of randomisation(s); details of any restriction (such as blocking and block size)** | **16** |
| **Allocation**  **concealment**  **mechanism** | **9** | **Mechanism used to implement the random allocation sequence (such as sequentially numbered containers), describing any steps taken to conceal the sequence until interventions were assigned** | **16** |
| **Implementation** | **10** | **Who generated the random allocation sequence, who enrolled participants, and who assigned participants to interventions** | **16** |
| **Blinding** | **11a** | **If done, who was blinded after assignment to interventions (for example, participants, care providers, those assessing outcomes) and how** | **n/a** |
|  | **11b** | **If relevant, description of the similarity of interventions** | **n/a** |
| **Statistical methods** | **12** | **Methods used to address each pilot trial objective whether qualitative or quantitative** | **21** |
| **Results** | | | |
| **Participant flow (a diagram is strongly recommended)** | **13a** | **For each group, the numbers of participants who were approached and/or assessed for eligibility, randomly assigned, received intended treatment, and were assessed for each objective** | **22 - 24** |
|  | **13b** | **For each group, losses and exclusions after randomisation, together with reasons** | **22** |
| **Recruitment** | **14a** | **Dates defining the periods of recruitment and follow-up** | **17** |
|  | **14b** | **Why the pilot trial ended or was stopped** | **n/a** |
| **Baseline data** | **15** | **A table showing baseline demographic and clinical characteristics for each group** | **24** |
| **Numbers analysed** | **16** | **For each objective, number of participants (denominator) included in each analysis. If relevant, these numbers**  **should be by randomised group** | **27 - 28** |
| **Outcomes and estimation** | **17** | **For each objective, results including expressions of uncertainty (such as 95% confidence interval) for any**  **estimates. If relevant, these results should be by randomised group** | **27 - 28** |
| **Ancillary analyses** | **18** | **Results of any other analyses performed that could be used to inform the future definitive trial** | **30** |
| **Harms** | **19** | **All important harms or unintended effects in each group (for specific guidance see CONSORT for harms)** | **n/a** |
|  | **19a** | **If relevant, other important unintended consequences** | **n/a** |
| **Discussion** | | | |
| **Limitations** | **20** | **Pilot trial limitations, addressing sources of potential bias and remaining uncertainty about feasibility** | **35 - 37** |
| **Generalisability** | **21** | **Generalisability (applicability) of pilot trial methods and findings to future definitive trial and other studies** | **35 - 37** |
| **Interpretation** | **22** | **Interpretation consistent with pilot trial objectives and findings, balancing potential benefits and harms, and**  **considering other relevant evidence** | **30 - 38** |
|  | **22a** | **Implications for progression from pilot to future definitive trial, including any proposed amendments** | **37 - 38** |
| **Other information** | | |  |
| **Registration** | **23** | **Registration number for pilot trial and name of trial registry** | **2** |
| **Protocol** | **24** | **Where the pilot trial protocol can be accessed, if available** | **11** |
| **Funding** | **25** | **Sources of funding and other support (such as supply of drugs), role of funders** | **39** |
|  | **26** | **Ethical approval or approval by research review committee, confirmed with reference number** | **16** |

**Citation: Eldridge SM, Chan CL, Campbell MJ, Bond CM, Hopewell S, Thabane L, et al. CONSORT 2010 statement: extension to randomised pilot and feasibility trials. BMJ. 2016;355.**

***We strongly recommend reading this statement in conjunction with the CONSORT 2010, extension to randomised pilot and feasibility trials, Explanation and Elaboration for important clarifications on all the items. If relevant, we also recommend reading CONSORT extensions for cluster randomised trials, non-inferiority and equivalence trials, non-pharmacological treatments, herbal interventions, and pragmatic trials. Additional extensions are forthcoming: for those and for up to date references relevant to this checklist, see** [**www.consort-statement.org**](http://www.consort-statement.org)**.**

**Table S2** (a) Baseline (time 1) and time 2 informant report of child symptoms pre and post phase 1 intervention for whole sample and (b) Time 1 and time 2 informant report of child symptoms for subgroups who were randomly allocated to each Phase 2 treatment, those who were not eligible for phase 2 treatment and those eligible who were not randomised.

|  | (a) Phase 1 intervention | | (b) Subgroups defined by eligibility for random allocation to phase 2 intervention;  mean (SD) pre and post phase 1 intervention | | | | | | | |
| --- | --- | --- | --- | --- | --- | --- | --- | --- | --- | --- |
|  | Whole sample; mean (SD)  (N=102) | | Not eligible after phase 1 treatment; mean (SD) (N=28) | | Eligible but not randomised; mean (SD) (N=12) | | RICAP; mean (SD)  (N=31) | | TAU; mean (SD)  (N=31) | |
|  | Time 1 | Time 2 | Time 1 | Time 2 | Time 1 | Time 2 | Time 1 | Time 2 | Time 1 | Time 2 |
| % PMT sessions  attended | 55.6  (32.1) | _ | 61.0  (29.8) | _ | 48.4  (31.1) | _ | 50.9  (32.5) | _ | 58.2  (34.4) | _ |
| Parent  CBCL externalising | 28.7  (12.2) | 23.5 **  (13.1) | 17.3  (8.4) | 10.4  (5.1) | 33.3  (10.6) | 26.7  (11.9) | 33.9  (11.3) | 30.1  (11.0) | 32.74  (8.8) | 28.80  (11.8) |
| SDQ conduct | 5.7  (2.4) | 4.4 **  (2.4) | 4.0  (1.8) | 2.1  (1.0) | 6.17  (2.7) | 4.73  (2.0) | 6.71  (2.1) | 5.39  (2.0) | 6.19  (2.2) | 5.63  (2.4) |
| Teacher  CBCL externalising | 17.3  (15.7) |  | 8.2  (8.7) | 6.0  (6.0) | 23.8  (17.9) | 18.6  (17.0) | 21.6  (18.8) | 18.2  (16.7) | 19.31  (13.4) | 19.93  (14.3) |
| SDQ conduct | 2.6  (2.5) | 2.3 *  (2.3) | 1.3  (1.7) | 1.0  (1.2) | 3.0  (2.3) | 2.1  (1.8) | 3.3  (3.1) | 2.7  (2.8) | 3.03  (2.2) | 3.14  (2.6) |

*, ** Paired t-test assessing whole group change pre (time 1) to post phase 1 (time 2) parent management training intervention indicated significant reduction in symptom reports at p < 0.05; p < 0.001 level respectively.

**Table S3:** Clinically reliable change following phase 2 intervention: Proportion of cases within clinical, borderline and normal ranges on parent and teacher report measures at each time point for children in the RICAP and TAU treatment arms

| Measure | Intervention | Assessment Time | Number and proportion (%) scoring in each score range on scale | | | | | | |
| --- | --- | --- | --- | --- | --- | --- | --- | --- | --- |
|  |  |  | Parent report | | | | Teacher report | | |
|  |  |  | Normal | Borderline | Clinical |  | Normal | Borderline | Clinical |
| SDQ  Conduct | RICAP | Before N = 31  After N = 28 | 1 (3.2)  1 (3.6) | 3 (9.7)  6 (21.4) | 27 (87.1)  21 (75.0) | N = 30  N = 27 | 18 (60.0)  14 (55.6) | 3 (10.0)  2(7.4) | 9 (30.0)  9 (37.0) |
|  | CTAU | Before N = 30  After N = 30 | 3 (10.0)  4 (13.7) | 2 (6.7)  4 (13.8) | 25 (83.3)  22 (72.5) | N = 29  N = 28 | 11 (37.9)  16 (57.1) | 5 (17.2)  4 (14.3) | 13 (44.8)  8 (28.6) |
| SDQ total problems | RICAP | Before N = 31  After N = 28 | 2 (6.5)  5 (17.9) | 8 (25.8)  6 (21.4) | 21 (67.7)  17 (60.7) | N = 30  N = 27 | 15 (50.0)  9 (33.3) | 5 (16.7)  7 (25.9) | 10 (33.3)  11 (40.7) |
|  | CTAU | Before N = 30  After N = 30 | 4 (13.3)  9 (31.0) | 4 (13.3)  3 (10.3) | 22 (73.3)  18 (58.6) | N = 29  N = 28 | 12 (41.4)  18 (64.3) | 3 (10.3)  3 (10.7) | 14 (48.3)  7 (25.0) |
| CBCL externalising | RICAP | Before N = 31  After N = 28 | 2 (6.5)  10 (32.1) | 2 (6.5)  3 (10.7) | 27 (87.1)  15 (57.1) | N = 30  N = 27 | 15 (50.0)  11 (40.7) | 2 (6.7)  6 (22.2) | 13 (43.3)  10 (37.0) |
|  | CTAU | Before N = 30  After N = 30 | 4 (13.3)  5 (17.2) | 2 (6.7)  2 (6.9) | 24 (80.0)  23 (75.9) | N = 29  N = 28 | 11 (37.9)  13 (46.4) | 4 (13.8)  4 (14.3) | 14 (48.3)  11 (39.3) |
| CBCL internalising | RICAP | Before N = 31  After N = 28 | 11 (35.5)  16 (60.7) | 5 (16.1)  5 (17.9) | 15 (48.4)  7 (21.4) | N = 30  N = 27 | 22 (73.3)  15 (55.6) | 3 (10.0)  3 (11.1) | 5 (16.7)  9 (33.3) |
|  | CTAU | Before N = 30  After N = 30 | 11 (36.7)  10 (34.5) | 8 (26.7)  4 (13.8) | 11 (36.7)  16 (51.7) | N = 29  N = 28 | 20 (69.0)  22 (78.6) | 7 (24.1)  1(3.6) | 2 (6.9)  5 (17.9) |
